# Evaluating the impact of MEDLINE filters on evidence retrieval: study protocol

**DOI:** 10.1186/1748-5908-5-58

**Published:** 2010-07-20

**Authors:** Salimah Z Shariff, Meaghan S Cuerden, R Brian Haynes, K Ann McKibbon, Nancy L Wilczynski, Arthur V Iansavichus, Mark R Speechley, Amardeep Thind, Amit X Garg

**Affiliations:** 1 Division of Nephrology, University of Western Ontario, London, Ontario, Canada; 2 Department of Medicine, McMaster University, Hamilton, Ontario, Canada; 3 Department of Clinical Epidemiology and Biostatistics, McMaster University, Hamilton, Ontario, Canada; 4 Department of Epidemiology and Biostatistics, University of Western Ontario, London, Ontario, Canada

## Abstract

**Background:**

Rather than searching the entire MEDLINE database, clinicians can perform searches on a filtered set of articles where relevant information is more likely to be found. Members of our team previously developed two types of MEDLINE filters. The 'methods' filters help identify clinical research of high methodological merit. The 'content' filters help identify articles in the discipline of renal medicine. We will now test the utility of these filters for physician MEDLINE searching.

**Hypothesis:**

When a physician searches MEDLINE, we hypothesize the use of filters will increase the number of relevant articles retrieved (increase 'recall,' also called sensitivity) and decrease the number of non-relevant articles retrieved (increase 'precision,' also called positive predictive value), compared to the performance of a physician's search unaided by filters.

**Methods:**

We will survey a random sample of 100 nephrologists in Canada to obtain the MEDLINE search that they would first perform themselves for a focused clinical question. Each question we provide to a nephrologist will be based on the topic of a recently published, well-conducted systematic review. We will examine the performance of a physician's unaided MEDLINE search. We will then apply a total of eight filter combinations to the search (filters used in isolation or in combination). We will calculate the recall and precision of each search. The filter combinations that most improve on unaided physician searches will be identified and characterized.

**Discussion:**

If these filters improve search performance, physicians will be able to search MEDLINE for renal evidence more effectively, in less time, and with less frustration. Additionally, our methodology can be used as a proof of concept for the evaluation of search filters in other disciplines.

## Background

We live in the information age, and the practice of medicine is increasingly complex and specialized. The conclusion that medical professionals have unmet information needs is inescapable[1-6]. Studies confirm opportunities to improve patient care[7-12]. Unfortunately, physicians are often unaware of new clinically relevant information and frequently report the need for supplementary information for patient encounters[13-17]. The amount of useful knowledge continues to grow, and is greater than any one practitioner can easily retain. Over the last decade, the MEDLINE database grew by over seven million citations, to 18 million citations[18-20] (as of May 2010). About 2,000 to 4,000 new references are now added each day[21].

### Finding practice evidence is a challenge

Traditional ways physicians acquired medical evidence have included reading textbooks, talking to colleagues, and subscribing to a select number of journals[3,22,23]. While these sources of information continue to be used, all have their challenges. Many textbooks are outdated by the time they are printed[24]. Colleagues frequently have the same challenge keeping up to date as the physician asking the question[25]. Best evidence may be widely dispersed across journals that are not typically reviewed. For example, articles relevant to the care of renal patients are published across 466 journals in over 18 different disciplines[26]. For this reason more and more physicians turn to the internet as a way to track down medical information[27-29]. Over 60% of physicians now have access to the internet in their clinical setting[29-31]. PubMed was introduced to the medical community in 1997[32]. This service provides free online access to the MEDLINE database.

### MEDLINE: promise and pitfalls

MEDLINE/PubMed is now the most widely used and accepted repository of medical literature, with over 1.3 billion searches performed in 2009[18]; it has been estimated that 15% the searches are conducted by physicians (personal communication D. Benson, National Library of Medicine staff). There is no doubt that PubMed has improved information management by health professionals[33-35]. However, searching MEDLINE can be time consuming and frustrating for many physicians[36]. In a laboratory setting, with no external pressures and time limits, it has been noted that health professionals spend, on average, half an hour per search topic to find, read, and critically appraise retrieved literature[37]. While, in practice, physicians only have time to spend an average of two minutes or less to find literature they need[1,38]. In truth, busy physicians shy away from literature searching in their daily routine. Since its inception, limitations to finding relevant studies in MEDLINE have been well documented[39].

**Figure 1:**
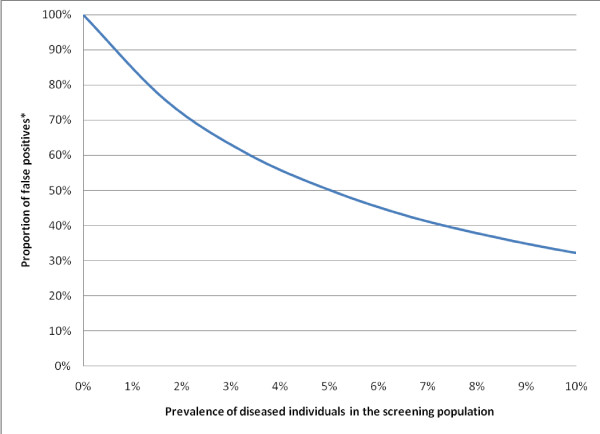
**Performance of a Diagnostic Tool with Sensitivity & Specificity of 95%**. A diagnostic tool with a high sensitivity and specificity results in a substantial proportion of false positives (among individuals with a positive test) when the prevalence of diseased individuals in the screening population is low; as the prevalence increases, the proportion of false positives decreases. *Proportion of False Positives: Proportion of individuals with a positive test who do not have the disease = (number of false positives)/(number of true positives + number of false positives).

Searching for relevant articles among large quantities of literature is akin to screening for rare diseases in populations. Even with an excellent screening tool with high sensitivity (ability to produce a positive test among people with disease) and high specificity (ability to produce a negative test among people without disease), screening a population in which the number of diseased individuals is low will result in identifying many false positives (a positive test for people without disease); see Figure 1 for an example. To curtail such findings, in clinical practice, screening of this nature is conducted on high-risk groups and not the entire population. For example, mammograms and colonoscopy procedures are often limited to higher-risk individuals over the age of 40. Using lessons learned from clinical practice, a potential solution to improve performance is to search portions of the bibliographic databases where relevant material is more likely to be present. A promising way to achieve this is to use filters that 'weed-out' unwanted information, leaving a higher concentration of relevant articles for searching.

### A solution to improve MEDLINE search performance: filters

The two most prominent performance metrics of literature searching are recall (also called sensitivity) and precision (also called positive predictive value; Table 1). Recall refers to the proportion of relevant articles retrieved from a set of relevant articles, while precision indicates the proportion of relevant articles retrieved from all the articles retrieved from a search. In other words, a small precision value means a lot of non-relevant articles have been retrieved.

**Table 1:** Formulae for calculating search recall, precision, and specificity

		**Relevant article**	**Non-relevant article**
		
*Search*	Articles found	a	b
	
	Articles not found	c	d

**Recall **(also referred to as **sensitivity **in diagnostic test terminology) a/(a+c), the proportion of relevant articles that were found by the search compared to the total number of relevant articles.

**Precision **(also referred to as **positive predictive value **in diagnostic test terminology) a/(a+b), the proportion of relevant articles that were found by the search compared to the total number of articles found by the search.

**Specificity **d/(b+d), proportion of articles not found by the search that are not relevant.

In an attempt to improve these two metrics for clinical users, our team and others have developed MEDLINE filters to enhance searching[40,41]. By selecting a filter for use, a clinical user is no longer searching the entire MEDLINE database; rather they are searching within a set of articles enriched for what they were looking for. Filters are, in essence, search strings optimized to retrieve all articles in MEDLINE for a given purpose (different purposes described below). To develop a filter, search terms are combined in various ways and formats using a systematic approach, and performance is measured. The terms (*e.g*., medical subject headings (MeSH), subject heading explosions, free-floating subheadings, heading words, and free text words) make special use of features provided when searching in MEDLINE, such as various search fields, Boolean operators, truncations, and wildcards. Depending on the topic, over a million MEDLINE filters can be tested to find the one that optimizes searching performance for a given purpose.

Members of our team previously developed and performed testing of two types of MEDLINE filters ('methods' and 'content')[42,43]. Testing was done by comparing filter performance against a hand search where research assistants categorized and assessed each article. Two forms of each type of filter were developed: narrow and broad. The narrow form yielded the highest specificity, while the broad form yielded the highest sensitivity (Table 1).

The first type of filter identifies articles of high methodological rigor for the prevention or treatment of health disorders, independent of any clinical discipline[43] ('methods' filter; Table 2). The best performing methods filters are currently a part of the PubMed interface, and can be accessed through the 'Clinical Queries' section[44]. The second type of filter identifies articles relevant to the practice of renal medicine[42] ('content' filter). We recently developed two high performance filters for this purpose (Table 3). Each of these filters reduces the MEDLINE database to sets of articles where information of interest is likely to be present. For example, applying the narrow renal 'content' filter to PubMed reduces the number of citations from 19,806,554 to 453,319 (when applied 15 May 2010). Given their promise, these MEDLINE filters now require further evaluation to determine their true benefits.

**Table 2:** Two high performance Methods filters for questions of therapy

Filter Form	PubMed Search Query
Broad	((clinical[Title/Abstract] AND trial[Title/Abstract]) OR clinical trials[MeSH Terms] OR clinical trial[Publication Type] OR random*[Title/Abstract] OR random allocation[MeSH Terms] OR therapeutic use[MeSH Subheading])

Narrow	(randomized controlled trial[Publication Type] OR (randomized[Title/Abstract] AND controlled[Title/Abstract] AND trial[Title/Abstract]))

PubMed fields: '*' = truncation character, [MeSH Terms] = exploded and focused MeSH term

**Table 3:** Two high performance renal Content filters

Filter Form	PubMed Search Query
Broad	"kidney diseases"[mh] OR "renal replacement therapy"[mh] OR renal[tw] OR kidney*[tw] OR (nephre*[tw] OR nephri*[tw] OR nephroc*[tw] OR nephrog*[tw] OR nephrol*[tw] OR nephron*[tw] OR nephrop*[tw] OR nephros*[tw] OR nephrot*[tw]) OR proteinuria[tw]

Narrow	("renal replacement therapy"[majr] OR "kidney diseases"[majr] OR kidney*[ti] OR nephr*[ti] OR renal[ti] OR "kidney"[majr:noexp] OR "renal dialysis"[mh] OR "kidney function tests"[majr] OR "proteinuria"[majr:noexp] OR glomerul*[ti]) NOT ("kidney neoplasms"[majr] OR pyelonephritis[majr:noexp] OR "urinary tract infections"[majr] OR "nephrolithiasis"[majr])

PubMed fields: '*' = truncation character, [majr] = exploded and focused MeSH term, [majr:noexp] = not exploded and focused MeSH term, [tw] = text word present in title, abstract or MeSH, [ti] = term present in title, [mh] = exploded MeSH term.

### The next stage in evaluation: whether the filters improve real physician searches

A search of the literature has identified no formal studies that evaluate the use of search filters by end-users. Thus, using key recommendations from reviews of information retrieval systems and search filters[39,40], we developed a testing framework that consists of six stages (Table 4).

**Table 4:** Search filter testing framework

Development	Stage one	Promising search filters are developed through a rigorous process of combining terms in various ways. The relevance of each article in a set of articles is defined a reference standard. The ability of a filter to restrict the set of articles to those that are relevant is then considered.
Validation	Stage two	Promising filters are independently evaluated on a second, distinct, set of articles to ensure equivalent performance in replication.

**Physician search performance**	**Stage three**	**Determine whether search filters improve end-user searching performance (i.e., recall and precision)**.

Physician knowledge	Stage four	Determine whether search filters improve physician knowledge.

Medical decisions or care	Stage five	Determine whether the acquired knowledge changes medical decision making or processes of care.

Patient outcomes	Stage six	Determine whether patient outcomes are improved.

To date, we have developed, optimized, and validated our filters in closed, experimental environments (stage one and two). The next stage is to determine if these MEDLINE filters improve physician's real searches (stage three). The efficient acquisition of medical evidence by physicians is essential to guide medical decision-making and patient care; this signifies a key step in the practice of evidence-based medicine[45]. Physician information management for patient care will improve if these filters can maximize the number of relevant articles retrieved, and minimize the number of non-relevant articles retrieved. This will enable physicians to search MEDLINE more effectively, in less time, and with less frustration.

Here we present our methodology for testing the aforementioned filters that was funded by a Canadian Institutes of Health Research operating grant focused on health services research. While our evaluation will focus on the retrieval of renal medical evidence (the purpose of the 'content' filter), these methods provide a framework for the objective testing of search filters that can be applied to any medical field.

### Objectives

Our primary objective will be to determine if a physician's use of MEDLINE filters when searching improves the identification of clinically relevant articles for a specific clinical question compared to their search unaided by any filters. Two types of filters, 'content' and 'methods,' will be tested either alone or together, resulting in eight different filter combinations.

### Specific Questions

1. Which filter combinations improve search recall the most?

2. Which filter combinations improve search precision the most?

3. Which filter combinations maximally optimize both search recall and precision?

### Hypotheses

The use of filters will improve a physician's search compared to an unaided search. A combination of both types of filters, 'content' and 'methods,' will produce the largest improvement in search recall and precision.

Literature searches can result in thousands or even hundreds of thousands of hits -- far too many for physicians to review. It would also be beneficial to know whether filters can improve the search results within a limited window of articles that physicians are most likely to review. The primary analysis focuses on all retrieved articles. In an additional analysis we will restrict the search results from PubMed to a cut-off level beyond which most physicians would no longer review citations (such as the top 60 citations).

## Methods

### Overview

The study is described in three steps:

1. We will assemble a series of clinical questions, to which there are a known set of relevant articles in MEDLINE.

2. We will survey nephrologists (kidney physicians), and ask each nephrologist what they would type in MEDLINE to find articles for a given clinical question.

3. We will determine the performance of each physician search, and how MEDLINE filters change the performance of each search.

We will use three methods to avoid bias and maximize generalizability: 1) we will use recently published systematic reviews to assemble the questions and identify sets of relevant articles. We will select those systematic reviews that detail reliable and comprehensive methods of assembling relevant articles for a focused clinical question. Using the included studies of these reviews will help ensure all sound evidence is accounted for, minimizing subjectivity in the selection of relevant studies. 2) we will use random rather than convenience sampling to select Canadian nephrologists for survey participation. We have already developed the survey using recommended survey design methods [46]. Our pilot test has proved we can obtain a high response rate. 3) when testing the impact of filter usage, we will adjust the alpha level of significance to avoid detecting spurious associations (type I errors) through multiple statistical comparisons.

### Step one: Assembling clinical questions and relevant articles Clinical questions

The search questions we pose need to be applicable to our main target user -- nephrologists. To assemble a representative set of clinical questions, we will use recently published renal systematic reviews. These reviews tend to target clinical questions for which uncertainty exists. Reviews will be gathered from EvidenceUpdates http://plus.mcmaster.ca/evidenceupdates. The EvidenceUpdates service provides a listing of systematic reviews from over 120 journals that meet rigorous methodological criteria[47]. EvidenceUpdates uses the following criteria to identify reviews: 'the clinical topic being reviewed must be clearly stated; there must be a description of how the evidence on this topic was tracked down, from what sources, and with what inclusion and exclusion criteria'[47,48]. To test the impact of the two treatment methods filters, we will only focus on questions of prevention and therapy. Two assessors will use a standardized checklist to independently confirm whether each review is pertinent to the care of renal patients. Assessors will be calibrated against a nephrologist in their application of checklist criteria. This method previously resulted in agreement beyond chance (kappa statistic), of 0.98[42]. Two assessors will further determine whether each review asks a focused clinical question with one main objective. To identify the clinical question to be used, we will abstract the primary objective of each review. Each objective will be transformed into a question (see example below), using the exact wording of each review. We will record all data abstracted for each systematic review in a standardized form. We will record the date for which information was compiled in each review, so that we can limit the subsequent MEDLINE searches to the appropriate start and end dates.

### Example

Objective: 'We aimed to assess whether prophylactic use of acetylcysteine reduces incidence of contrast nephropathy in patients with renal insufficiency.'[49]

Clinical Question: Does prophylactic use of acetylcysteine reduce the incidence of contrast nephropathy in patients with renal insufficiency?

### Relevant articles

The purpose of performing a MEDLINE search is to identify relevant articles for the question of interest. For the current study, we require a set of relevant articles in MEDLINE for each clinical question. Instead of using a subjective measure of relevance, we will deem the primary articles included in each review and also indexed in MEDLINE as relevant. Well-conducted systematic reviews use a variety of comprehensive methods to identify all high-quality primary studies for a particular clinical question. This will help ensure the articles used in our analysis are sufficiently important using an external standard. Primary articles included in the systematic reviews but not indexed in MEDLINE, such as commentaries, abstracts, books, or theses will be excluded, as will journal articles not indexed in MEDLINE. To determine if an article is available in MEDLINE, we will abstract the title, primary author, year of publication, and journal title for each article. MEDLINE will be accessed through the PubMed interface http://www.pubmed.gov. One assessor will use the PubMed single citation matcher tool to search for each article. If the article is present, the article's unique identifier will be recorded. A random sample of 10% of the articles will be searched for in duplicate by a second, independent, assessor to determine searcher-reliability. The second assessor will also confirm that each collected PubMed identifier corresponds to the proper extracted citation.

### Step two: Surveying nephrologists

This study will use real search queries created by nephrologists in Canada. We developed a survey that asks nephrologists to enter a search query for MEDLINE that they would use to answer a pre-specified clinical question. To minimize respondent burden, each nephrologist will only receive a single, unique clinical question. Because knowledge on how physicians search for medical information is, in general, very limited, we also expanded the survey to acquire key data on their information-gathering practices and use of the internet. The survey will also ask respondents to self-report the number of results that they generally scan per search; this will aid in our secondary analysis which restricts the search to a cut-off level beyond which most physicians no longer search (for example, the survey could establish that physicians stop after the first 60 citations). The survey was pilot tested for validity and usability by three academic and two community-based nephrologists. The survey was approved by the research ethics board at the University of Western Ontario.

Using the Royal College of Physicians and Surgeons of Canada[50], Provincial Colleges of Physicians and Surgeons[51] and the Canadian Medical Directory[52] online databases, we have identified 519 practicing academic and community nephrologists in Canada. The survey will be conducted in a random sample, applying the tailored design method outlined by Dillman[46]. All surveys will be coded to track non-responders. We will initially contact each nephrologist by email (if available) or by phone to determine if they will participate in our survey. For interested participants, the survey will be sent using the modality of preference (email, fax or mail). Online or paper-based versions of the survey will be made available for each interested participant. If a response is not received in two weeks, a follow-up correspondence will be sent. If a response is still not received three weeks later, a fourth correspondence will be attempted. Records will be kept of the number of non-respondents.

### Step three: Testing filters

For each clinical question we will perform nine different searches. The first search will use terms provided by a physician, unaided by any filters. The next eight searches will combine the terms provided by a physician with at least one type of filter ('methods' or 'content') (Table 5). The nine searches reflect three options for each of the 'methods' and 'content' filters (no filter, broad filter or narrow filter), for a total of three (methods) × three (content) = nine different searches, or one physician search and eight different filter combinations.

**Table 5:** Filters available for testing

Category	Available Filters	Special Instructions
Methods[43] (therapy)	Broad filter Narrow filter	Remove all methods terms from physician-generated search query

Content[42]	Broad filter Narrow filter	Remove all renal content terms from physician-generated search query

Some physicians may submit search queries with misspelled terms or phrases, which may result in the retrieval of no citations. In some cases adding in a filter will similarly result in no citations being retrieved. Alternatively, the benefits of filters may be exaggerated if the misspelled word is replaced by the filter. To avoid this issue in the primary analysis, where necessary, the syntax of physician provided search queries will be modified slightly. A list of modification rules is provided below. All modifications will be conducted independently and in duplicate by two assessors and any discrepancies in decisions will be resolved by consensus. To determine if the findings are robust, we will look for consistency of results in additional analyses where we will test the searches provided by physicians without any modifications.

### Rules for syntactically improving physician provided search queries

1. Update MeSH terms indicated as exploded terms and add PubMed syntax for limits described

2. Correct spelling errors

3. Capitalize Boolean terms (AND, OR, NOT)

4. Remove commas ',' periods '.' semi-colons ';' and apostrophes "'"

5. Replace '/' with an OR term

6. Replace 'and/or' with an OR term

7. Replace '+' with an AND term

8. Remove preposition and article terms (e.g. 'in,' 'by,' 'at,' 'for,' 'from,' 'a,' 'the')

9. Expand short forms or acronyms and include the original term with an OR term

The use of filters for subject areas (methods or renal information) is advantageous, as some terms need not be entered in the search query. Rather, the filters act as a substitute for certain terms. For example, instead of adding the term 'clinical trial' to a search query, a user can simply select the methods filters, which would limit MEDLINE to those studies using best methods for questions of therapy (*i.e*., randomized clinical trials). Thus, when we add the methods and/or renal content filters to physician searches, we will need to remove any methods and/or renal content terms in the physician's search query. To do so, each search query will be reviewed independently and in duplicate by two assessors trained in epidemiology and by two assessors trained in medicine. Discrepancies in decisions to remove terms by the assessors will be resolved by consensus.

### Example

Clinical Question: What are the benefits of intradermal compared to intramuscular hepatitis B vaccination in chronic kidney disease?

Search query provided by a physician: hapititis b vaccination dialysis randomized trial

Modified search query as per listed rules: hepatitis b vaccination dialysis randomized trial

Query aided by methods filter: hepatitis b vaccination dialysis AND <methods filter>

Query aided by content filter: hepatitis b vaccination randomized trial AND <content filter>

Query aided by methods and content filter: hepatitis b vaccination AND <methods filter> AND <content filter>

Due to the large number of PubMed searches required (9 searches × 100 clinical questions = 900 searches), the searching process will be automated through the use of the E-utilities resource available from PubMed[53]. We have tested this process and confirmed that the results retrieved through E-utilities match those retrieved using the PubMed interface. For each search, we will collect the total number of articles retrieved and the number of relevant articles retrieved. To determine the latter, we will compare the PubMed unique identifiers of the retrieved articles to the PubMed identifiers of the relevant articles identified from the systematic review for the specified clinical question. We will restrict each search to the search dates provided in the methods section of each systematic review; date restriction will be used to exclude articles, both relevant and non-relevant, that could not have been included in the systematic review process.

### General statistical analytic strategy, sample size, and sensitivity analyses

#### Primary analysis

We will calculate differences in recall between every physician's unaided search, and the physician's searches when each of eight filter combinations is applied. We will use a two-sided one-sample (paired) t-test for each filter combination to determine if a difference exists (Null Hypothesis, H_0_: mean difference in recall between unaided search and search with filter = 0, Alternate Hypothesis, H_1_: mean difference in recall not = 0). We will then rank the performance of each filter combination that enhanced the unaided search, and examine this list descriptively. We may perform additional post-hoc t-tests amongst top performing filter combinations to determine which combination was the best. We will then repeat this entire statistical process for the outcome precision. Filter combinations that improve both recall and precision (best-performing filter combinations) will be examined descriptively. A large number of significance tests will be conducted in this study (eight tests for recall, eight tests for precision, total 16 tests). To reduce the risk of type I error, we will apply the conservative method of Bonferroni so that tests with a p < 0.003 will be interpreted as statistically significant[54].

#### Secondary analysis

We will use the responses provided by nephrologists to determine the number of results that three-quarters of the respondents do not scan beyond. This number will be rounded to the closest multiple of 20. A value of 20 is used because it reflects one page of search results in PubMed on the default setting. For example, if 75% of the respondents indicate they do not look beyond 52 results, we would use 60 as a cut-point to signify three search pages in PubMed. This secondary analysis will be identical to the primary analysis except that we will calculate the values of recall and precision limited to citations within the defined cut-point. For example, for a cut-point of 60 results, the measures would be calculated for articles retrieved in the first 60 results (or three default pages of results).

#### Other analysis

We will analyze the baseline characteristics of non-responding physicians, compared to physicians who do respond, to elucidate systemic non-response and aid with conclusions of generalizability.

#### Sample size

We expect to identify 100 systematic reviews that meet our criteria. Using our pilot data, we estimate a standard deviation of 0.23 for the difference in recall, and a standard deviation of 0.34 for the difference in the precision. Given a sample of 100 clinical question responses (with each nephrologist receiving a single unique question) power of 80% and a significance p-value of 0.003, using a two-sided one-sample t-test, we will have the ability to detect a minimum of 9.0% mean difference in recall and a 13.2% mean difference in precision between a filtered search and an unaided search. These values represent a reasonable benefit to warrant the ongoing effort to incorporate the filters into use. Sample size calculations were performed using SAS Statistical Package version 9.1 (SAS Institute Inc., Cary, NC, and U.S.A.).

#### Sensitivity analyses

In the primary analysis of this study, we will consider each article listed in a systematic review as equally important. However we recognize that some articles may be considered more important and influential than others, and a searcher may be most interested in identifying these seminal articles. To address this point, we will perform sensitivity analyses to test whether filters help identify the most important articles, as defined by two different criteria as outlined below.

##### Criterion one: Articles referenced in UpToDate

This analysis will focus on the articles listed in the systematic reviews that are referenced in UpToDate. For each review, two assessors will independently conduct a search in UpToDate using the objective statement of the review as a guide. The assessors will document the entries that cover the review topic; each search may recover several UpToDate entries. All entries will be compiled and an assessor will evaluate each entry to determine whether included studies from the review were referenced; each referenced article will be tagged as an important article for the current analysis. Finally, systematic review topics not covered in UpToDate will be excluded from analysis.

##### Criterion two: Highly cited articles

This analysis will focus on the top cited articles from each systematic review. For each article, we will search Web of Science to identify the number of times the article was cited by other publications. If Web of Science does not provide a citation count, we will then search Scopus. If Scopus fails to provide a citation count, we will search Google Scholar. If none of the sources provide a citation count, the article will be assigned a citation count of one because we are certain that the study was cited by at least one systematic review. After retrieving all citation counts, we will tabulate the median citation count for all articles included in each systematic review. Articles with citation counts greater than or equal the median value will be tagged as important articles in each systematic review for the current analysis.

### Other considerations

#### Minimizing threats to validity

Our protocol has adapted methodology originating from the field of information retrieval. We have attempted to control for the following biases identified in previous studies on search engine evaluation[55,56]:

Suggestion: To ensure internal validity, a sufficiently large number of search topics must be used to produce meaningful evaluations of search engine effectiveness.

Solution: We will use 100 recently published systematic reviews in nephrology to assemble a variety of clinical questions and identify corresponding sets of relevant articles.

Suggestion: To ensure external validity, search topics should be motivated by the genuine information needs of the target users.

Solution: We will identify renal systematic reviews. Systematic reviews target questions for which uncertainty exists and are of interest to nephrologists.

Suggestion: To ensure external validity, search queries used to evaluate the retrieval quality should be derived by individuals in the target population.

Solution: Through the use of a survey, we will obtain search queries from practicing nephrologists.

Suggestion: To ensure overall validity, relevance judgments must be made in relation to the target population.

Solution: We will use the primary articles included in high quality systematic reviews to identify relevant literature. Through this procedure, we are engaging widely accepted principles of evidence-based medicine to identify the most important primary literature to retrieve in a search. We will select those systematic reviews that detail reliable and comprehensive methods of assembling relevant articles for a focused therapy question; this will help ensure all sound evidence is accounted for, minimizing subjectivity in the selection of relevant studies.

Furthermore, several other methods to avoid bias and maximize generalizations will be used:

1. To avoid misclassification of the outcome, we will record the date for which information was compiled in each review and subsequently limit all searches to the appropriate start and end dates. Date restriction will be used to preclude articles, both relevant and non-relevant, not considered in the systematic review process. In addition, we will only include primary studies that are indexed in PubMed.

2. By ensuring that each included systematic review targets only one objective, the study will further minimize misclassification by ensuring that all included articles in the review are truly relevant for the corresponding treatment question.

3. We will minimize selection bias by random, rather than convenience sampling, to select Canadian nephrologists for survey participation. This will ensure that a large variety of nephrologists with varied search abilities participate in the study. Clinical questions will be randomly assigned to each nephrologist ensuring that, on average, physicians have equal familiarity with the topic. We will also evaluate the characteristics of non-responding physicians to physicians for whom responses are received to identify potential systemic non-response that may impair the random nature of the responses.

4. For the survey, we will employ the tailored design method to maximize response[46].

5. When testing the impact of filter usage, we will adjust the alpha level of significance to avoid detecting spurious associations (type I errors) through multiple statistical comparisons.

6. We will employ a paired design to ensure equivalence in potential biases between the unaided and filter-aided searches.

### Additional considerations

#### Determining article relevancy

There is no perfect, easily applied measure to determine whether an article is relevant to a focused clinical question. We propose to use primary articles identified in systematic reviews, as an external measure of relevance in this study. All other articles will be viewed as non-relevant. We recognize there are additional articles, such as commentaries, narrative reviews, case reports, and animal studies which some may consider relevant. However, by using systematic reviews to define relevance, we are engaging widely accepted principles of the hierarchy of evidence to identify the most important articles to retrieve in a search. Also, our primary analytic method is a 'paired design' where we compare physician search performance with and without the use of filters. Any misclassification of article relevance is expected to impact all the queries in a similar manner, with no major effect on differences observed between search strategies.

#### Performance metrics

In this study, we will use recall and precision as metrics to determine how well a reference set of relevant articles are retrieved. Some may say this is a misleading surrogate outcome. We agree that other more relevant outcomes would be desired. For example, it would be useful to know whether the use of filters improves a physician's ability to come up with the correct answer (better knowledge), whether this changes medical decisions or processes of care, and whether this improves patient outcomes. The current study represents a key milestone in a staged program of research, to guide the development and execution of future studies (Table 4).

#### Systematic reviews focus on questions of therapy

Currently, most systematic reviews pertain to prevention and treatment. For reasons of feasibility, we are only testing methods filters related to therapy in this project. However, more systematic reviews for diagnosis, prognosis, and etiology are being published every day. This will allow us to reliably assess other methods filters in the future.

#### Searching is a dynamic process

The initial search queries we receive from physicians will be entered online, or received by mail. In truth, searching is a dynamic process -- an unsuccessful search is tried again using different terms. Also, what physicians report in a survey may differ from what they do in front of their own computers. We did consider a different research framework, such as video surveillance of local nephrologists using MEDLINE filters. However, for reasons of feasibility and generalizability, we propose to obtain the initial search queries from a random sample of nephrologists practicing in academic and non-academic settings across Canada. We are testing their first search. If filters substantially improve search performance we may obviate the need for additional searches, saving time and reducing frustration.

#### Target audience is nephrologists

We will focus on nephrologists for four reasons: we are testing content filters designed to identify articles relevant for the care of renal patients; subspecialists are frequently interested in identifying and reviewing primary studies for focused questions in their field; the systematic reviews identified through EvidenceUpdates database are primarily targeted at physicians; and we have access to a list of nephrologists in Canada. Proving the filters work with this audience will guide future evaluations with other health care workers and other disciplines.

## Summary

This project will test the performance of search filters on real physician searches. Here, we have outlined a detailed research plan that includes many measures to avoid bias. The challenge of finding medical evidence will only increase as the number of indexed citations increases. Our methodology can serve as a proof of concept for evaluating MEDLINE search filters in other subject areas and for other audiences. If our research can prove a positive impact of search filters on physician searching this may improve the MEDLINE searching of renal professionals worldwide. Our research is a key milestone in a staged program of research to guide future evaluations of MEDLINE filters on physician knowledge and uptake, medical decision making, and processes of care.

## Competing interests

The authors declare that they have no competing interests.

## Authors' contributions

This paper is based on the protocol submitted for peer review funding that included authors AXG, RBH, KAM, SZS and NLW as investigators. SZS wrote the initial draft of this manuscript and all other authors reviewed, provided feedback, and approved the final manuscript.
